# Reduced Graphene Oxide and Gold Nanoparticles-Modified Electrochemical Aptasensor for Highly Sensitive Detection of Doxorubicin

**DOI:** 10.3390/nano13071223

**Published:** 2023-03-30

**Authors:** Fanli Kong, Jinping Luo, Luyi Jing, Yiding Wang, Huayu Shen, Rong Yu, Shuai Sun, Yu Xing, Tao Ming, Meiting Liu, Hongyan Jin, Xinxia Cai

**Affiliations:** 1 State Key Laboratory of Transducer Technology, Aerospace Information Research Institute, Chinese Academy of Sciences, Beijing 100190, China; 2 School of Electronic, Electrical and Communication Engineering, University of Chinese Academy of Sciences, Beijing 100049, China; 3 Obstetrics and Gynecology Department, Peking University First Hospital, Beijing 100034, China

**Keywords:** doxorubicin, electrochemical detection, aptasensor, reduced graphene oxide, gold nanoparticles

## Abstract

Doxorubicin (DOX) is the most clinically important antibiotic in cancer treatment, but its severe cardiotoxicity and other side effects limit its clinical use. Therefore, monitoring DOX concentrations during therapy is essential to improve efficacy and reduce adverse effects. Here, we fabricated a sensitive electrochemical aptasensor for DOX detection. The sensor used gold wire as the working electrode and was modified with reduced graphene oxide (rGO)/gold nanoparticles (AuNPs) to improve the sensitivity. An aptamer was used as the recognition element for the DOX. The 5′ end of the aptamer was modified with a thiol group, and thus immobilized to the AuNPs, and the 3′ end was modified with methylene blue, which acts as the electron mediator. The combination between the aptamer and DOX would produce a binding-induced conformation, which changes the electron transfer rate, yielding a current change that correlates with the concentration of DOX. The aptasensor exhibited good linearity in the DOX concentration range of 0.3 μM to 6 μM, with a detection limit of 0.1 μM. In addition, the aptasensor was used for DOX detection in real samples and results, and showed good recovery. The proposed electrochemical aptasensor will provide a sensitive, fast, simple, and reliable new platform for detecting DOX.

## 1. Introduction

Cancer, also known as a malignant tumor, has become a severe public health problem and a primary cause of death worldwide with population growth and global aging [1,2,3]. The application of anticancer drugs is one of the most widely used therapeutic options [4,5]. Doxorubicin (DOX) is an anthracycline antibiotic with broad spectra of chemotherapeutic applications and anti-neoplastic applications [6,7,8], widely used in the treatment of lung cancer, liver cancer, Hodgkin lymphoma, acute leukemia, and many other types of cancer [9,10,11]. DOX facilitates cancer treatment through several mechanisms, such as the disruption of DNA replication by inserting DNA base pairs and the disruption of DNA repair by interacting with topoisomerase II [12,13,14,15,16]. However, the effects of DOX are not cancer cell-specific; it also affects the DNA functioning of normal cells, resulting in irreversible toxicity to organs such as the heart, brain, and kidneys, which can be life-threatening in severe cases [17,18]. Based on the dangerous side effects of DOX and the individual differences of patients, monitoring DOX levels in patients’ biological samples during treatment is vital to improve the effectiveness of therapy, reduce adverse effects, and promote personalized medicine [19,20,21].

As research proceeds, a variety of methods have emerged, greatly enriching the detection of DOX. Liquid chromatography [14,22], high-performance liquid chromatography [23,24], UV-Vis spectroscopy [25,26], capillary electrophoresis [27,28], and fluorescence spectroscopy [29,30] are currently the classical methods for the detection of DOX, and the detection limits can reach several nanomoles. However, some of these methods require sophisticated and complex sample processing, expensive equipment, extensive technical experience, and long measurement and analysis times. In contrast, others are poorly reproducible and lack sufficient sensitivity and selectivity [31,32]. Electrochemical methods have been recognized as an ideal alternative to other assays, with inexpensive equipment, simple operation, no complex pretreatment of biological samples, high selectivity, high sensitivity, and fast response times, which are well suited for the rapid clinical detection of DOX [9,33].

An electrochemical aptasensor is an essential analytical tool that uses aptamers as recognition elements, combined with electrochemical detection methods. The aptamer is an oligonucleotide sequence obtained by in vitro selection and has a binding affinity comparable to antibodies [34]. Aptamers also have many advantages that antibodies cannot match, such as a broader range of targets, lower production costs, better stability, and easier chemical modification, making them an increasingly crucial molecular tool in medical diagnostics [35,36]. For example, Sun and Xing et al. have achieved the detection of vascular endothelial growth factor C and programmed death-ligand 1 by exploiting the change of steric hindrance after target binding to aptamer [37,38]. Cao et al. utilized analyte-kissing-induced structure-switching aptamers to accomplish drug delivery and simultaneously achieve in vivo detection of interferon-γ (IFN-γ) [39]. There are few studies on electrochemical aptamer sensors for DOX, and most of them focus on the direct electrochemical detection of DOX, but this requires high oxidation potential and has limited anti-interference ability [40]. Therefore, developing a DOX detection tool based on electrochemical aptamers has excellent application prospects.

Due to their unique properties that are different from traditional materials, nanomaterials are making a splash in sensor modifications and can improve the analytical performance of sensors [41,42]. Graphene, a new material consisting of carbon atoms tightly stacked into a monolayer two-dimensional honeycomb structure [43], has been attracting widespread attention since it was first stably prepared by micromechanical exfoliation in 2004 [44]. Graphene has excellent electron mobility, extremely low resistivity, a large specific surface area, and good flexibility [45]. These unique mechanical and electrical properties make it widely used in the field of electrochemical sensing [46,47]. Gold nanoparticles (AuNPs) are a kind of metal nanomaterial with superior properties and are widely used for the modification of electrodes [48,49]. The large specific surface area of AuNPs allows more material to be immobilized, and its good conductivity improves the detection sensitivity of the sensor [50]. In addition, AuNPs have good biocompatibility and high surface activity and can interact with various groups to facilitate the immobilization of antibodies and aptamers [51].

In response, we have fabricated a highly sensitive and selective electrochemical sensor for the timely and rapid detection of DOX. The reduced graphene oxide (rGO)/AuNPs were used as a platform to improve the analytical performance of the sensor, and were modified onto the electrode surface by one-step electrochemical deposition. A published DOX aptamer was used as the recognition element, which is a hairpin structure with methylene blue (MB) acting as the signal molecule modification at the 3′ end. The combination of DOX and aptamer produces a binding-induced conformational change, leading to a change in the rates of electron transfer rates from MB at the end of the aptamer to the electrode. We evaluated the analytical performance of the DOX electrochemical aptasensor by detecting DOX in phosphate-buffered saline (PBS). We also assessed the repeatability and selectivity of the sensors, using the same method. Considering the advantages of simple, convenient, and sensitive electrochemical sensors, we are optimistic that this sensor will provide a promising platform for the timely and rapid detection of DOX.

## 2. Materials and Methods

### 2.1. Apparatus

We used Gamry Reference 600 electrochemical instruments (Philadelphia, PA, USA) to perform the chronocoulometry (CC) experiment. Other electrochemical experiments, including electrochemical impedance spectroscopy (EIS), cyclic voltammetry (CV) and square wave voltammetry (SWV), were carried out in an Autolab PGSTAT302N electrochemical workstation (Herisau, Switzerland). The deionized water used for the experiments was generated with the Michem ultrapure water apparatus (Chengdu, China) with a resistance of 18 MΩ/cm. Scanning electron microscope (SEM) images were obtained using a Hitachi S-3500 scanning electron microscope (Tokyo, Japan). A Hechuang KH2200E ultrasonic generator (Kunshan, China) was used in homogenizing the solution.

### 2.2. Reagents and Materials

The 6-mercapto-1-hexanol (MCH) was ordered from TCI Chemicals (Tokyo, Japan). Ethanol, potassium chloride (KCl), Potassium ferrocyanide trihydrate (K_4_Fe(CN)_6_), Sodium hydroxide (NaOH), and gold (III) chloride (HAuCl_4_) trihydrate were received from Sinopharm Chemical Reagent Co., Ltd. (Shanghai, China). Potassium ferricyanide (K_3_Fe(CN)_6_), tris-2-carboxyethyl-phosphine (TCEP), Tris (hydroxymethyl) aminomethane (Tris), and doxorubicin hydrochloride were purchased from Sigma Aldrich (Saint Louis, MO, USA). Graphene oxide solution (GO, 2 mg mL^−1^) was obtained from Xianfeng nanomaterials company (Nanjing, China). Hexaammineruthenium (III) chloride (RuHex), dacarbazine (DTIC), tetracycline hydrochloride (TET), and chloramphenicol (CPL) were obtained from Acmec Biochemical Co., Ltd. (Shanghai, China). The Tris-EDTA buffer (TE buffer, PH = 8.0) and phosphate-buffered saline (PBS, pH = 7.4) were purchased from Solarbio (Beijing, China). All of the reagents used in the work were of analytical grade and used as received.

The 200 μm diameter gold wire was ordered from Alfa Aesar (Ward Hill, MA, USA). Heat-shrink polytetrafluoroethylene insulation was obtained from ZEUS (Branchburg Township, CA, USA).

The DOX aptamer was chosen according to prior reported works in the literature [52] and synthesized by Sangon Biotechnology (Shanghai, China) with the following sequence: 5′-SH-(CH_2_)_6_-ACC-ATC-TGT-GTA-AGG-GGT-AAG-GGG-TGG-T-Methylene Blue-3′. Upon receipt, we diluted the aptamer concentration to 200 µM with TE buffer and stored it in 200 μL aliquots at −20 °C until use.

### 2.3. Fabrication of the Sensor Working Electrodes

Gold wire was cut into 3.5 cm lengths to make the sensor working electrode. The actual sensing window was 7 mm long and located at the front end of the gold wire. There was a 1.5 cm conductive part at the other end for the electrochemical instrument connection. The rest of the middle part was insulated with polytetrafluoroethylene tube. The insulated sensors were sonicated in 2 M NaOH solution, anhydrous ethanol solution, 2 M H_2_SO_4_ solution, and deionized water, in turn, for 10 min to initially remove impurities from the electrode surface. After that, the electrode was electrochemically cleaned by CV in 0.5 M H_2_SO_4_ solution. Electrochemical cleaning was performed on the gold wire at a scan rate of 100 mV s^−1^ over a voltage range of −0.35 V to 1.5 V for 8 to 10 cycles.

### 2.4. Preparation of rGO/AuNPs-Modified Electrode

The rGO/AuNPs composites were modified on the surface of the working electrode by a one-step electrochemical deposition technique to increase its surface area and obtain a larger response current. Our plating solution was obtained by mixing 2 mg mL^−1^ GO solution with 10 mg mL^−1^ HAuCl_4_ solution at a volume ratio of 9:1 and ultrasonicating it for 30 min. Then, CV was conducted in the potential ranges of −1.5 to 0.6 V for 15 cycles at a scan rate of 50 mV s^−1^.

### 2.5. Frabriction of DOX Aptasensor

The thiol group of the aptamer and the gold nanoparticles on the electrode formed a Au-S bond, thus fixing the aptamer to the electrode. Firstly, an aliquot of the DOX aptamer solution (200 μM, 2 μL) was thawed and then reduced with 10 μL of 100 μM TCEP solution for 1 h at room temperature to reduce the 5′-disulfide bone. Subsequently, the reduction-treated aptamer was diluted to 300 nM with PBS, and the rGO/AuNPs-modified electrode was immersed in the aptamer solution for 2 h at room temperature. Subsequently, the electrode was immersed in the MCH solution (20 mM) at 4 °C for 12 h to remove nonspecifically adsorbed DNA and produce a self-assembly well-aligned monolayer (SAM) of thiol modified aptamer. Then, the electrode was rinsed thoroughly with deionized water to remove residual MCH and stored in PBS at 4 °C for future use.

### 2.6. Electrochemical Measurements

A three-electrode system consisting of a nanomaterial-modified gold wire as the working electrode, a 0.6 mm diameter platinum electrode as the counter electrode, and a commercial Ag/AgCl (3 M KCl) electrode as the reference electrode was used in all electrochemical experiments. CC experiments used to calculate the electroactive areas of the working electrodes were carried out in 5 mM K_3_Fe(CN)_6_ solution containing 0.1 M KCl as the supporting electrolyte, with a pulse width of 1 V and a pulse period of 1 s. The other CC experiment, conducted in 10 mM Tris–HCl buffer (pH = 7.4) in the absence and presence of 50 μM RuHex, with a pulse width of 0.5 V and a pulse period of 0.5 s, was used to measure aptamer surface density. EIS measurements were performed in a 5 mM K_3_Fe(CN)_6_/K_4_Fe(CN)_6_ mixture containing 0.1 M KCl as the supporting electrolyte, with a frequency range from 10^5^ Hz to 0.1 Hz and a sinusoidal voltage perturbation of 5 mV. SWV measurements were carried out to obtain the response of the aptasensor to different concentrations of DOX. The SWV curves were performed in the PBS solution (PH = 7.4) with a potential range of −0.5 V to 0 V, a potential step width of 1 mV, a pulse amplitude of 50 mV, and a frequency of 300 Hz. It was worth noting that the DOX and aptasensor were allowed to incubate for 20 min before SWV measurement to allow the sufficient binding of DOX and aptamer. All the above experiments were carried out at room temperature.

## 3. Results

### 3.1. Characterization of rGO/AuNPs Nanomaterials and Constructed rGO/AuNPs/Apt Interface

**Scheme 1:**
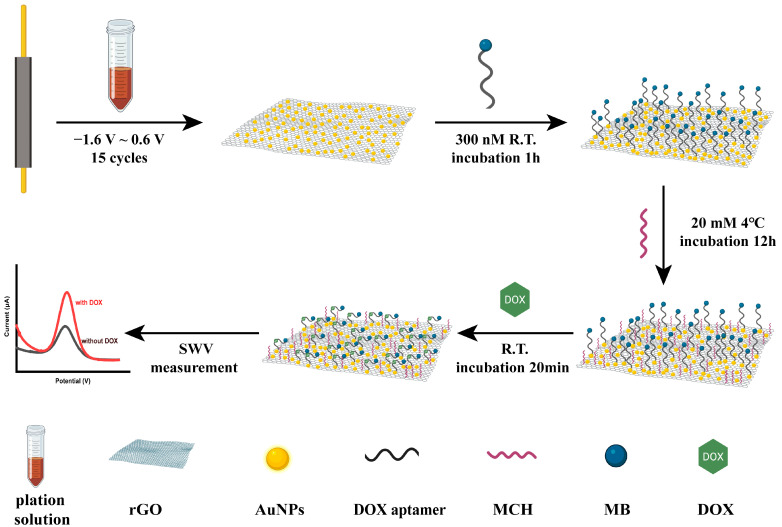
Step-by-step modification process of the electrochemical aptamer sensor, and the detection principle.

The working electrodes were modified with rGO/AuNPs nanomaterials for the sensitive and fast detection of DOX. The schematic diagram of the modification of the working electrode was shown in Scheme 1.

**Figure 1:**
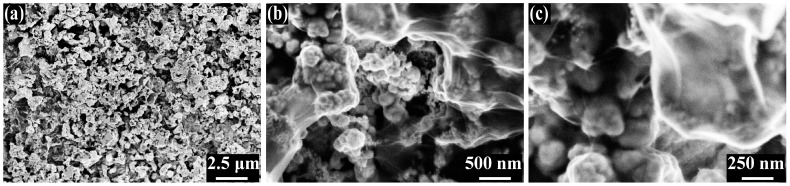
(**a**) The overall morphology of the electrochemically deposited rGO/AuNPs; (**b**,**c**) The magnified morphology of the outermost rGO/AuNPs.

The rGO/AuNPs were electrodeposited together on the electrode by CV. During the negative voltage scan, some oxygen-containing groups of GO in contact with the electrode were irreversibly reduced. The resultant rGO was less soluble, and thus directly attached to the electrode surface. At the same time, AuCl_4_^−^ was also reduced to obtain AuNPs. After electrodeposition, the rGO/AuNPs composite was formed on the electrode surface, and its SEM images are shown in Figure 1. Figure 1a shows the overall morphology of the electrochemically deposited nanomaterials. In the figure, the rGO/AuNPs nanocomposite shows a rough porous-like structure, which greatly increased the specific surface area of the electrode. Figure 1b,c shows the enlarged morphology of rGO/AuNPs; the outer layer of rGO was muslin-like, and the AuNPs, appearing spherical, attached to the wrinkled structure formed by the underlying rGO. The SEM image of the bare gold electrode is shown in Figure S1a,b. The surface of the electrode was flat and smooth, without any material attached to it. The unique morphology of rGO/AuNPs can immobilize more aptamers and accelerate electron transfer, thus amplifying the current signal. The energy dispersive X-Ray spectrometer (EDS) of the rGO/AuNPs-modified electrode is provided in Figure S1c.

**Figure 2:**
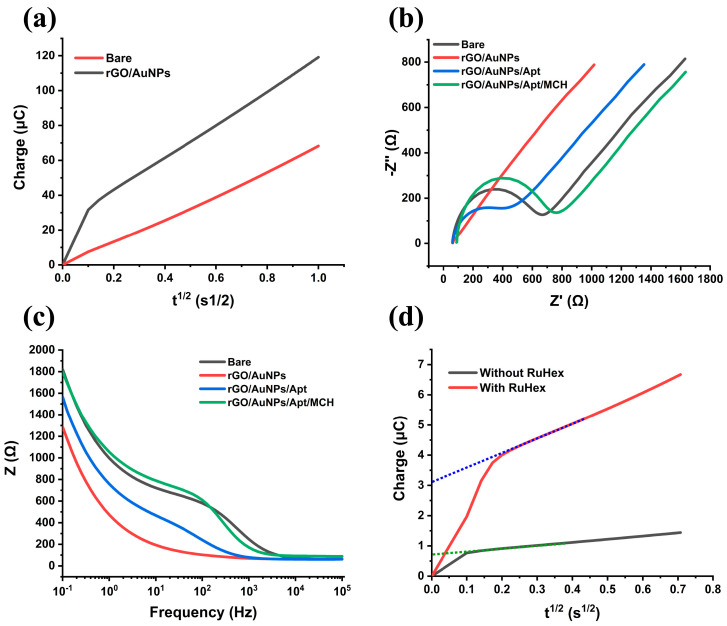
(**a**) CC responses of the bare electrode and rGO/AuNP modified electrode in 5 mM K_3_Fe(CN)_6_ solution containing 0.1 M KCl; (**b**) the Nyquist plot and (**c**) Bode plot of the bare electrode and rGO/AuNP modified electrode in 5 mM K_3_Fe(CN)_6_/K_4_Fe(CN)_6_ mixture; (**d**) CC responses of the aptasensor in the absence and presence of 50 μM RuHex.

CC experiments performed in 5 mM K_3_Fe(CN)_6_ solution containing 0.1 M KCl were used to evaluate the electroactive area change of the electrodes after rGO/AuNP modification. The experimental results are shown in Figure 2a. The area of the electrodes can be calculated using the Cottrell integrated equation:

Q = 2nFACD^1/2^t^1/2^π^−1/2^ (1)

where Q is the charge (C), n is the number of electrons transferred, F is the Faraday constant (96,485 C eq^−1^), A is the electrode area (cm^2^), C is the molar concentration of the active species (mol cm^−3^), D is the diffusion coefficient (7.6 × 10^−6^ cm^2^ s^−1^ for 5 mM K_3_Fe(CN)_6_ solution [53]), and t is time (s). There is a linear relation between Q and t^1/2^. According to the slope of the plot of Q vs. t^1/2^, we found that the electroactive areas of bare electrodes and rGO/AuNP modified electrodes were 0.049 cm^2^ and 0.065 cm^2^, respectively.

EIS was used to study the impedance changes during the stepwise modification of the electrode surface. The EIS responses of bare, rGO/AuNPs-modified electrodes, rGO/AuNPs/Apt, and the rGO/AuNPs/Apt/MCH interface in the 5 mM K_3_Fe(CN)_6_/K_4_Fe(CN)_6_ mixture are shown in Figure 2b,c. The Nyquist plot in Figure 2b consists of a semicircle at the high frequency and a linear section at the low frequency. The semicircle in the high frequency region represents the charge transfer impedance (R_ct_) of the electrode. The R_ct_ of the bare electrode and the rGO/AuNPs-modified electrode were 599.15 Ω and 58.24 Ω, respectively, indicating that the modification of nanomaterials enhanced the transfer of electrons between the electrode surface and electrolyte solution, thereby increasing the sensitivity of the sensor. When the rGO/AuNPs-modified electrode was successively incubated with DOX aptamer and MCH, the Rct increased to 415.93 Ω and 645.21 Ω, respectively, which was consistent with the principle, indicating that the sensor was successfully fabricated. The Bode plot of the electrode impedance was shown in Figure 2c. We focused on the impedance at 300 Hz, which was the frequency used at SWV. The impedances of bare, rGO/AuNPs-modified electrodes, rGO/AuNPs/Apt and the rGO/AuNPs/Apt/MCH interface were 444.2 Ω, 83.38 Ω, 131.50 Ω, and 374.83 Ω, respectively, which were consistent with the changing trends of R_ct_, indicating the successful fabrication of the sensor from the other aspects.

### 3.2. Determination of Aptamer Surface Density

According to the previously reported literature [54,55], the CC measurements were performed using RuHex acting as a redox marker to determine the aptamer surface density. The general principle is as follows: the negatively charged DNA phosphate skeleton and the positively charged RuHex have electrostatic attraction, and the density of the aptamer can be calculated according to the amount of charge required for the reduction of RuHex absorbed on the electrode surface, with the following equation:

Γ_apt_ = (Q_ab_N_A_/nFA)(z/m) (2)

where Γ_apt_ is the aptamer surface density (molecules cm^−2^), Q_ab_ is the charge required for the reduction of RuHex adsorbed on the electrode surface (C), N_A_ is the Avogadro’s number, n is the number of electrons transferred, F is the Faraday constant (96,485 C eq^−1^), A is the electroactive area of the electrode (cm^2^), z is the charge of RuHex (z = 3), and m is the number of bases of the aptamer (m = 28). According to the difference between the intercept of the linearly fitted plot in the presence and absence of RuHex (Figure 2d), which refers to the Q_ab_, the aptamer surface density of the rGO/AuNPs-modified electrode was 2.37 × 10^13^ molecules cm^−2^.

### 3.3. Electrochemical Properties of the Aptasensor

**Figure 3:**
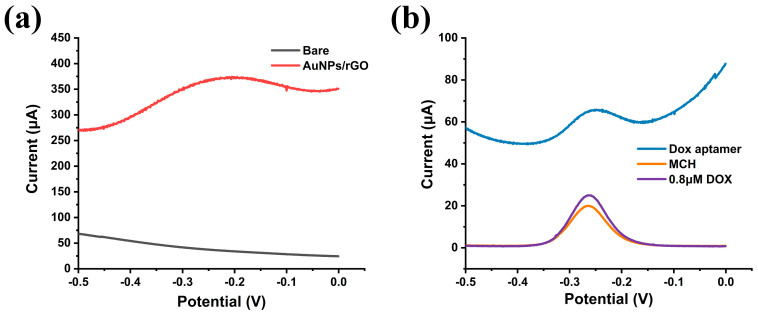
(**a**) SWV responses of bare electrodes and rGO/AuNPs-modified electrodes; (**b**) SWV responses of electrodes incubated with Dox aptamer, MCH, and 0.8μM DOX, respectively.

The properties and stepwise modification of the working electrode were tested through SWV in PBS. Bare and rGO/AuNPs-modified electrodes had no response in the voltage range of −0.5 V to 0 V (Figure 3a). This was because MB had not been fixed to the electrode. After the aptamer was immobilized to the electrode, we observed a significant SWV response near −0.25 V, which was caused by the oxidation of MB modified on the aptamer (Figure 3b). At this time, despite having a high peak current, the current baseline was also very high. However, when the aptasensor was incubated with MCH afterwards, the peak SWV current of the sensor was reduced and the current baseline was also reduced with it. This may be due to the formation of a well-aligned SAM of the thiol-modified aptamers.

The detection principle of the sensor was shown in Scheme 1. When DOX was not binding to the aptamer, the aptamer was in a “stretched” state and the MB at the end of the aptamer was far away from the electrode surface. When the aptamer was bound to DOX, the conformation of the aptamer changed to form a “folded” state, so that the MB was close to the electrode surface, increasing the rates of electrons transferred from the MB to the electrode. When we used SWV to detect the DOX, we could obtain an increased peak current. As shown in Figure 3b, after combining with DOX the peak currents of the SWV response increased from 20.02 μA to 24.97 μA, in accordance with our detection principle.

### 3.4. Analytical Results of the DOX Aptasensor

**Figure 4:**
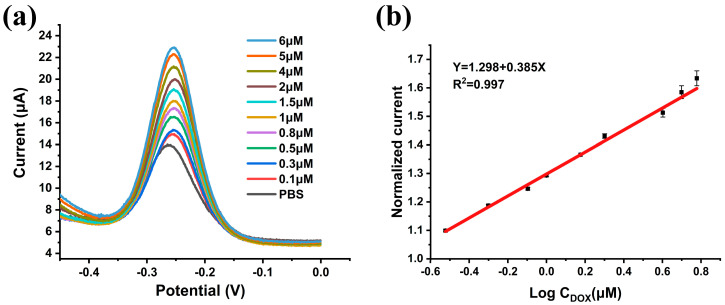
(**a**) SWV responses of the aptasensor to different concentrations of DOX standard solutions (0.1 μM to 6 μM) in 0.1 M PBS solution; (**b**) calibration curve of the aptasensor between DOX concentration and normalized SWV peak current.

We measured the SWV response of the aptasensor to standard DOX samples at different concentrations in PBS (PH = 7.4) to evaluate the detection performance of the aptasensor. The binding event of the aptamer to the target does not involve the transfer of electrons, which means that the current changes we detected arose exclusively from changes in electron transfer rates from MB immobilized at the end of the aptamer to the electrode. As shown in Figure 4a, the peak current of the SWV response rose with increasing DOX concentration, consistent with our detection principle. To eliminate the differences between sensor batches, we normalized the SWV peak current and obtained the relationship between the normalized current and the DOX concentration. As shown in Figure 4b, the normalized SWV peak current (I_norm_) and log concentration of DOX (C_DOX_) showed good linearity over the range of 0.3 μM to 6 μM for DOX, and the linear response can be described as I_norm_ = 1.298 + 0.385 Lg C_DOX_ (μM). The correlation coefficient was 0.996, and the limit of detection was 1 μM. We fitted the sensor’s signal gain to the Langmuir isotherm and calculated the apparent dissociation constant to be 2.55 uM. A list of recently developed electrochemical sensors for DOX detection is presented in Table 1. Our sensor had a more balanced performance, and its detection range basically covered the therapeutic range of drugs in human blood [56].

### 3.5. Evaluation of Repeatability, Selectivity and Stability of the Aptasensor

**Figure 5:**
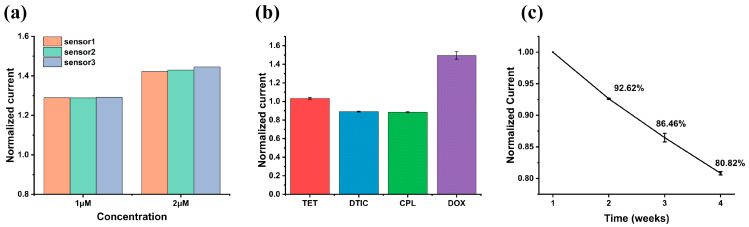
(**a**) Normalized SWV currents of 3 different aptasensors responding to 1 μM and 2 μM DOX were used to evaluate the repeatability of the sensors; (**b**) normalized SWV current responses of aptasensors to 50 μM of TET, DTIC, CPL, and 4 μM DOX, respectively; (**c**) stability of the proposed sensor for three weeks.

Reproducibility is an essential evaluation indicator of sensor performance. We evaluated the reproducibility of the aptasensor by measuring the SWV response of three independent sensors in solutions containing DOX. The responses of the aptasensors in 1 μM and 2 μM DOX are shown in Figure 5a. Their normalized peak currents were 1.290, 1.289, and 1.292 for 1 μM DOX, and 1.423, 1.430, and 1.446 for 2 μM DOX, respectively. Based on the above experimental results, the coefficients of variation were calculated to be 0.11% and 0.81%, respectively, indicating the excellent repeatability of the aptasensor.

Selectivity is another important characteristic of the aptasensor. We measured the SWV response of the sensor to 50 μM of TET, DTIC, CPL, and 4 μM of DOX, respectively, to evaluate the selectivity of the sensor. The results are shown in Figure 5b. The normalized current responses of the sensor to the interferents were 3.37%, −10.96%, and −11.48%, respectively, which were much smaller than 49.55% of DOX, indicating the excellent selectivity of the aptasensor.

In addition, we also investigated the stability of this aptasensor. The sensor was soaked in PBS (PH = 7.4) and stored in a refrigerator at 4 °C. We measured the aptasensor’s SWV response in PBS solution (PH = 7.4) weekly. The results are shown in Figure 5c. After three weeks, compared with the first week, the sensor’s SWV peak current decreased to 80.32% of the original.

### 3.6. Real Sample Analysis

Normal human serum samples were used to evaluate the performance of the aptasensor in real samples. The serum was diluted 10 times with PBS (PH = 7.4), and the DOX sample at a determined concentration was added. The spiked human serum samples were measured using the proposed aptasensors. As is shown in Table 2, the recoveries of the sensors in human serum samples ranged from 94.8% to 109.0%, indicating that the aptasensor has the potential for clinical application.

## 4. Conclusions

In this work, we fabricated an rGO/AuNPs-modified aptamer sensor for the electrochemical detection of DOX. Compared with previous research, this work has the following highlights: (1) rGO/AuNPs were successfully modified on the working electrode by one-step electrochemical deposition. They enlarged the surface area and decreased the electron transfer impedance of the electrode, thus improving the sensitivity of the sensor. (2) The signal of the sensor came from the conformational change of DOX binding to the aptamer, so it can reduce the interference caused by the non-specific adsorption of other substances. (3) No other reagents needed to be added during the assay, which makes the operation simple and convenient. We believe that this new aptamer sensor can provide a timely, convenient, simple, and sensitive platform for the detection of DOX.

## Figures and Tables

**Table 1:** Comparison of our work with the recently published electrochemical method for the determination of DOX.

Electrode	Linear Range (nM)	Detection Limit (nM)	Reference
CPE/CoFe_2_O_4_/MWCNTs	0.05–1150	0.01	[57]
Gold electrode/aptamer	31–125	28	[58]
Microfluidic chip/aptamer	0.01–10	10	[59]
GCE/PR/GO/Fe_3_O_4_/K	60–950	8	[31]
GCE/GQD	18–3600	16	[60]
Gold electrode/AuNPs/antibody	0.0084–0.294	0.00153	[61]
BBD	5–50	1.63	[62]
Gold wire/rGO/AuNPs/aptamer	300–6000	100	This work

CPE: carbon paste electrode; MWCNTs: multiwalled carbon nanotubes; GCE: glassy carbon electrode; PR: polyrhodanine; GO: graphene oxide; K: kombucha solvent; GQD: graphene quantum dot; BBD: boron-doped diamond electrode.

**Table 2:** Results of aptasensor determination of DOX in spiked human serum samples.

Sample	Added (μM)	Detected (μM)	Recovery (%)
Human serum	0.5	0.474	94.8
	1	1.071	107.1
	2	2.180	109.0

## Data Availability

Not applicable.

## Supplementary Materials

The following are available online at https://www.mdpi.com/article/10.3390/nano13071223/s1, Figure S1: (**a**,**b**) Scanning electron microscope (SEM) images of bare electrode at different scales; (**c**) the energy dispersive X-Ray spectrometer (EDS) of the rGO/AuNPs-modified electrode.
