# Supplementary material for: Reduced Graphene Oxide and Gold Nanoparticles-Modified Electrochemical Aptasensor for Highly Sensitive Detection of Doxorubicin
Source: Nanomaterials (Basel). 2023 Mar 30;13(7):1223. doi: 10.3390/nano13071223 (PMC10096947; doi:10.3390/nano13071223)
Supplement: Supplementary file 1 [file nanomaterials-13-01223-s001.zip › nanomaterials-2287381-supplementary.pdf]

nanomaterials

Supplementary Materials

Reduced Graphene Oxide and Gold Nanoparticles-Modified Electrochemical Aptasensor for Highly Sensitive Detection of Doxorubicin

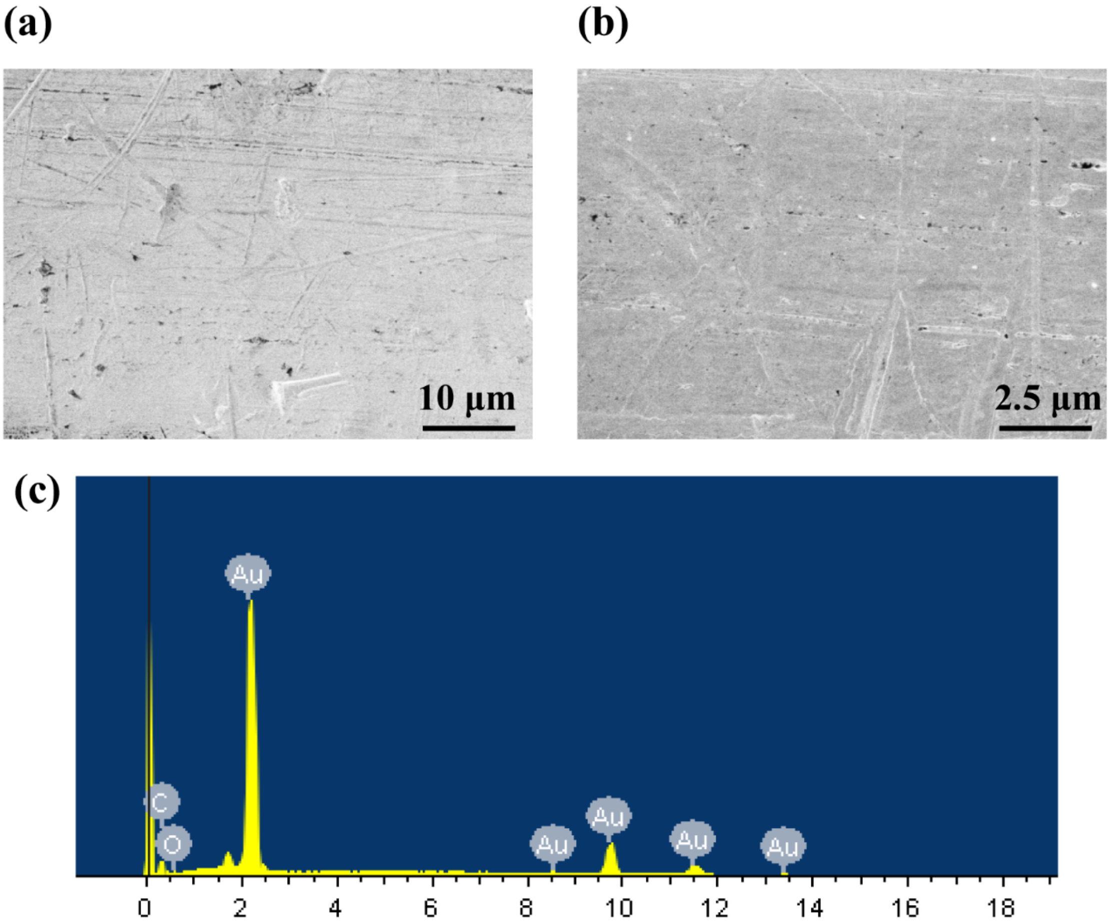

Figure S1. (**a**,**b**) Scanning electron microscope (SEM) images of bare electrode at different scales; (**c**) The energy dispersive X-Ray spectrometer (EDS) of the rGO/AuNPs modified electrode.
